# An integrative approach for a network based meta-analysis of viral RNAi screens

**DOI:** 10.1186/s13015-015-0035-7

**Published:** 2015-02-13

**Authors:** Sandeep S Amberkar, Lars Kaderali

**Affiliations:** Institute of Medical Informatics and Biometry, Medical Faculty, TU Dresden, Fetscherstr. 74, Dresden, 01307 Germany; ViroQuant Research Group Modeling, BioQuant, Heidelberg University, INF 267, Heidelberg, 69120 Germany; Present address: Department of Translational Genomics/Center for Molecular Medicine, University of Cologne, Robert-Koch Str. 21, Cologne, 50931 Germany

**Keywords:** Network analysis, RNAi screening, Virus-host interactions

## Abstract

**Background:**

Big data is becoming ubiquitous in biology, and poses significant challenges in data analysis and interpretation. RNAi screening has become a workhorse of functional genomics, and has been applied, for example, to identify host factors involved in infection for a panel of different viruses. However, the analysis of data resulting from such screens is difficult, with often low overlap between hit lists, even when comparing screens targeting the same virus. This makes it a major challenge to select interesting candidates for further detailed, mechanistic experimental characterization.

**Results:**

To address this problem we propose an integrative bioinformatics pipeline that allows for a network based meta-analysis of viral high-throughput RNAi screens. Initially, we collate a human protein interaction network from various public repositories, which is then subjected to unsupervised clustering to determine functional modules. Modules that are significantly enriched with host dependency factors (HDFs) and/or host restriction factors (HRFs) are then filtered based on network topology and semantic similarity measures. Modules passing all these criteria are finally interpreted for their biological significance using enrichment analysis, and interesting candidate genes can be selected from the modules.

**Conclusions:**

We apply our approach to seven screens targeting three different viruses, and compare results with other published meta-analyses of viral RNAi screens. We recover key hit genes, and identify additional candidates from the screens. While we demonstrate the application of the approach using viral RNAi data, the method is generally applicable to identify underlying mechanisms from hit lists derived from high-throughput experimental data, and to select a small number of most promising genes for further mechanistic studies.

**Electronic supplementary material:**

The online version of this article (doi:10.1186/s13015-015-0035-7) contains supplementary material, which is available to authorized users.

## Background

RNA interference (RNAi) has become an important workhorse of functional genomics, and genome-wide RNAi screens have been employed for example to identify genes involved in cell growth and viability, proliferation, differentiation, signaling or trafficking [1-9]. The technology has furthermore accelerated the discovery of novel host dependency factors (HDF) and host restriction factors (HRF) in viral infection [10-19]. However, while RNAi is a very powerful tool to identify genes involved in a specific biological process, the placement of hits in their functional and spatiotemporal context in the underlying molecular processes remains a major challenge [20,21]. The interpretation of RNAi data in particular for virus screens is complicated further by the observed low overlap between identified host factors, even in different screens targeting the same virus [22-24]. This low overlap has been explained by different experimental conditions such as host cell type and viral strain used, transfection, incubation and infection time, and siRNA library used [24] as well as by technical artifacts arising from cell population context [25,26]. Furthermore, due to the typical setup of RNAi experiments with primary screens followed by secondary validation assays, it is likely that published hit lists are highly specific, but not very sensitive, further explaining the low overlap observed between different screens at the level of individual genes [27]. This, however, severely restricts a comparative analysis of inter-species RNAi screens [28]. On the other hand, protein interaction networks, virus-host interaction networks and other heterogeneous data have increased tremendously [29-34]. This offers novel ways to interpret hit lists from RNAi experiments from a network perspective, by integrating individual hits in their systemic context. It has been shown that this approach increases the overlap between different screens for the same virus at the pathway level [24], and the method can be extended to meta-analysis of screens targeting different viruses. Being less dependent on individual genes, but rather focusing on pathways, may shed new light onto virus-specific and generic host processes facilitating or restricting infection, and may prove a promising approach to identify potential host targets for antiviral drug development.

Several meta-analyses of RNAi screens have been conducted, albeit most work focused on integrating different screens targeting a single virus [24,28,35,36]. A notable exception is the study by Snijder et al., including 45 screens targeting 17 different mammalian viruses [37]. The authors show that accounting for cellular heterogeneity improves gene overlaps between screens, but the study does not focus on functional regions within the host protein network targeted by different viruses. In contrast, Navratil et al. study virus-host protein interactions in the human interferon network [32], throwing light on how viruses of different families target the innate immune system. Other similar analyses focused largely on HIV, for example, Murali et al. employed a semi-supervised machine learning approach mapping RNAi hits onto a protein interaction network to predict new HDFs [38]. Macpherson et al. and similarly Maulik et al. mine the HIV-1 human protein interaction network using biclustering, and identify biclusters enriched with GO terms and RNAi hits [39,40]. Several authors have furthermore used protein-protein interaction (PPI) networks to identify topological properties of proteins targeted by pathogens. Dyer et al. characterized host proteins targeted by 190 different pathogens, including 35 viruses, 17 bacterial and two protozoan groups [29]. One of the major outcomes of this analysis was that pathogens preferentially target proteins with high node betweenness (bottlenecks) or high degree (hubs). Similarly, the studies by Dijk et al. and Dickerson et al. both showed that HIV preferentially targets hub and bottleneck genes in the human protein network [30,31]. Further characterizing the neighborhood of HDFs, Gulbahce et al. showed that proteins translated from genes involved in viral diseases are most likely located in the neighborhood of their corresponding viral targets [33].

Given the typically low overlap between different RNAi screens at the gene level and the relatively long hit lists resulting from individual screens, a central problem is how to select most promising candidates for functional characterization and detailed biochemical follow-up experiments. When looking for putative antiviral drug targets, one is typically interested in candidates that have a significant impact on infection outcome in the specific virus under consideration, or possibly even in several different viral species if e.g. broadly acting antivirals are sought for. Corresponding target pathways should therefore be “enriched” by hit genes from the RNAi data, while at the same time it is desirable that the respective targets are centrally located in the virus-host interaction network.

In this manuscript, we present a comparative analysis of RNAi hits for different viruses in the context of functional modules of protein interaction networks. The main purpose of our work is in hit prioritization, that is, we strive to identify a small set of candidates for further detailed follow-up experiments. We cluster the host protein network to identify functional host modules, and then use a statistical test to identify modules enriched with hits from seven genome-wide RNAi screens for three different viruses. Network topological characteristics are used to filter relevant subnetworks further, and resulting modules and their neighborhoods are annotated and interpreted. Using this approach, we identified several interesting candidate pathways for human immunodeficiency virus 1 (HIV-1) and hepatitis C virus (HCV), including known targets such as the mediator complex or members of the heterogeneous nuclear ribonucleoprotein subunits (hnRNPs) in HIV infection, or MAP kinases and heat shock proteins in HCV infection. Furthermore, using our approach, we predict that SERCA1 and Tankyrase-1 (TNKS1) may be interesting targets for further characterization in HCV infection.

## Materials and methods

**Figure 1:**
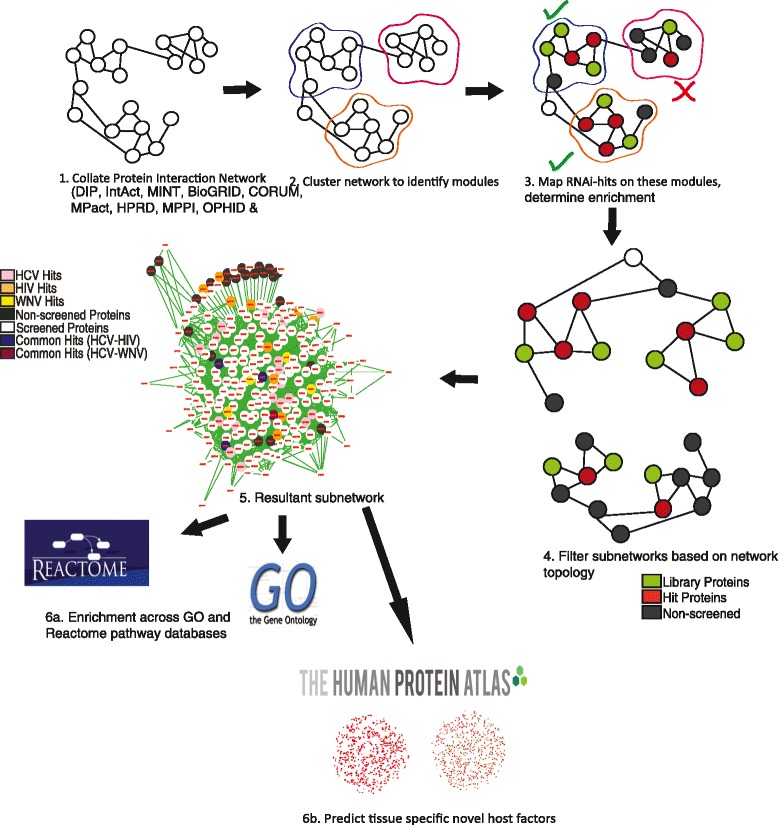
**Overview of the data analysis pipeline.** **(1)** Protein interactions from public databases are collated to build an integrated human PPI network. **(2)** Greedy unsupervised clustering is used to identify relevant, possibly overlapping, submodules in the PPI network. **(3)** Hits from one or several RNAi screens are mapped to these modules and modules are filtered for significant enrichment. **(4)** Subnetworks are further filtered based on network topology and semantic similarity values. **(5)** Resulting modules are visualized as subnetworks, color-coded for hits, non-hits, and **(6a,b)** are then functionally characterized based on GO and Reactome pathway. **(6c)** Lastly, using gene expression data from different tissues, tissue-specific putative novel host factors are predicted.

An overview of the data analysis pipeline used is shown in Figure 1. In brief, we collate information from 11 different public protein-protein interaction (PPI) data repositories, and integrate them into a large human PPI network. Subsequently, we use a cohesiveness-based greedy clustering algorithm to identify –possibly overlapping– clusters in the protein network, which are then tested for enrichment of hits from one or several RNAi screens. Significant modules are then filtered further using topological properties and semantic similarity, and functionally characterized using gene ontology and Reactome pathways. Using tissue-specific expression data, we predict novel putative host factors based on neighborhood relations in identified modules. We describe each of these steps in more detail in the following.

### Human protein interaction network:

The human protein interaction network was collated from two major resources: the iRefIndex database, a meta-database comprising data from ten resources (DIP, IntAct, MINT, BioGRID, BIND, CORUM, MPact, HPRD, MPPI, OPHID [41-51]), and the String v9.0 database [52] which includes both experimentally validated as well as computationally predicted interactions. The union of reported interactions in these databases was used to establish our PPI network. We utilized a score filter of 0.75 on the STRING interactions as a tradeoff between reliability of included interactions and sufficient network density for further computations. Different thresholds between 0.6 and 0.9 were tested for the predicted interactions from STRING. For higher scores, the predicted interactions did not add much to the existing pool of interactions, and subsequent clustering resulted in few to no subnetworks. Conversely, for lower scores, the subnetworks included broad networks with multiple, non-specific functional annotations. A score of 0.75 led to optimal subnetworks that were functionally specific, and returned a reasonable number of subnetworks for further analysis. The overall procedure resulted in a protein interaction network comprising 15,383 proteins and 337,413 interactions from STRING and iRefIndex.

### RNAi screening data:

We then mapped data from seven published genome-wide RNAi screens to the PPI network, including three human immunodeficiency virus-1 (HIV-1) screens [10,12,53], three Hepatitis C Virus (HCV) screens [13,18,54] and one west Nile virus (WNV) screen [11]. Further data analysis was then performed individually using only screens targeting the same virus (intra-species), as well as across all seven screens (inter-species).

### Submodule identification and statistical testing:

We used the ClusterONE algorithm to detect overlapping subnetworks in the human PPI network. ClusterONE is a neighborhood-expansion, greedy graph clustering algorithm [55]. It is able to take edge weights corresponding to confidence scores into account in the clustering, and allows overlapping clusters where individual proteins may be part of more than one cluster. We used default values for most parameters of the ClusterONE algorithm, except for the merge-method parameter which was set to multi to merge highly overlapping clusters, as well as the minimum cluster size parameter, which we varied between 25 and 100. The variation of the cluster size parameter leads to clusters of different granularity, from very small, highly cohesive clusters, to larger and more heterogeneous clusters. Both may be desirable for the analysis of virus-targeted subnetworks, we therefore continued analysis with a redundant set of larger and smaller, overlapping clusters; we label this set of clusters **C**^*all*^ in the following. Note that these clusters are not merged or integrated further, but rather **C**^*all*^ is a set of different clusters. After clustering, we tested for significant enrichment of RNAi hits within each cluster in **C**^*all*^ using Fisher’s exact test, with significance level *α*=0.05, resulting in the set **C**^*hit*^⊂**C**^*all*^ of clusters significantly enriched with RNAi hits. We note that the clusters in **C**^*hit*^may still overlap and may even contain clusters that are subsets/supersets of one another.

### Submodule filtering and cluster selection:

We next used additional filtering criteria to select a small number of relevant clusters from **C**^*hit*^ for further manual analysis. The underlying idea is to choose clusters that differ significantly from non-significant clusters not only based on their enrichment with RNAi hits, but also with respect to their “importance” in the underlying host PPI network. We selected seven network centrality measures and two further similarity measures for this filtering step. We briefly review these measures in the following, but before repeat some elementary definitions from graph theory.

Let *G*=(*V*,*E*) be an undirected graph with nodes *v*∈*V* corresponding to proteins and undirected edges *e*∈*E* corresponding to interactions between proteins. As we consider undirected edges only, let *e*_*i*,*j*_=*e*_*j*,*i*_. We define a *pathP* between two nodes *s*,*t*∈*V* in a graph *G*=(*V*,*E*) as a sequence *v*_0_, *e*_0_, *v*_1_, *e*_1_,..., *v*_*k*−1_, *e*_*k*−1_, *v*_*k*_ of nodes *v*_*i*_∈*V* and edges *e*_*i*_∈*E*, where edge *e*_*i*_ connects nodes *v*_*i*_ and *v*_*i*+1_, where *v*_*i*_≠*v*_*j*_ for all nodes in *P*, and where *v*_0_:=*s* and *v*_*k*_:=*t*. The length of *P* is defined as the number of edges in the path *P*.

When clustering the graph *G* using a graph clustering algorithm such as ClusterONE, the nodes *V* in *G* are grouped into different clusters. Let *V*_*C*_⊆*V* be one such cluster. This cluster induces a subnetwork *S*_*C*_=(*V*_*C*_,*E*_*C*_) on *G*, where *E*_*C*_={*e*_*i*,*j*_∈*E*:*v*_*i*_,*v*_*j*_∈*V*_*C*_}, i.e., the induced subnetwork consists of the subset *V*_*C*_ of nodes, and all edges in *E* between these nodes in the original graph *G*. Hereafter, we use the term *subnetwork* to denote the full subnetwork *S*_*C*_=(*V*_*C*_,*E*_*C*_), whereas by *cluster* we refer only to the subset of nodes *V*_*C*_⊆*V*.

To filter significant clusters *V*_*C*_∈**C**^*hit*^ further, we used the following topological properties of the nodes in *V*_*C*_ respectively their induced subnetwork *S*_*C*_:

1. **Average node degree:** The *node degree* of a vertex *v* in a graph *G*=(*V*,*E*) is given by
  $$\text{deg}(v,G) := | \{e_{v,w}\in E \quad | \quad \forall w \in V \} |, $$
  i.e., it is the number of edges in *E* adjacent to *v*. The *average node degree* of a subnetwork *S*_*C*_=(*V*_*C*_,*E*_*C*_) of *G* is the average degree of all nodes in *V*_*C*_:
  $$C_{D}(S_{C})=\frac{1}{|V_{C}|}\sum\limits_{v \in V_{C}} \text{deg}(v,S_{C}), $$
  where |*V*_*C*_| denotes the number of nodes in *V*_*C*_. Note that we compute the degree with respect to the edge set *E*_*C*_ of the subgraph *S*_*C*_, and not the full graph *G*.
2. **Average node betweenness:** The *node betweenness* of a node *v*∈*V* is the ratio of the number of shortest paths between any two nodes *s, t* in *G* that pass through *v*, to the total number of shortest paths between any two nodes in *G*. Let *Ψ*(*v*) be the set of ordered pairs (*s,t*) in *V*×*V*, so that *s*, *t* and *v* are distinct. Then,
  $$C_{B}(v,G)=\sum\limits_{(s,t) \in \Psi(v,G)}\frac{\sigma(s,t|v,G)}{\sigma(s,t|G)}, $$
  where *σ*(*s*,*t*|*G*) is the total number of *s,t*-shortest paths in *G*, and *σ*(*s*,*t*|*v*,*G*) is the number of shortest paths from *s* to *t* in *G* that pass through node *v*. The *average node betweennessC*_*B*_(*S*_*C*_) of a subgraph *S*_*C*_ is the average node betweenness of all nodes *v*∈*V*_*C*_ in the subgraph *S*_*C*_,
  $$C_{B}(S_{C})=\frac{1}{|V_{C}|}\sum\limits_{v \in V_{C}} C_{B}(v,S_{C}). $$
3. **Average node closeness:** The normalized *closeness* of a node *v*∈*V* is defined as
  $$C_{Clo}(v,G)=\frac{1}{|V|-1}\left(\sum\limits_{w \in V, w \neq v}d(v,w|G)\right)^{-1}, $$
  where *d*(*v*,*w*|*G*) is the length of the shortest path between two nodes *v*,*w*∈*V*. The *average node closenessC*_*Clo*_(*S*_*C*_) of a subgraph *S*_*C*_=(*V*_*C*_,*E*_*C*_) is
  $$C_{Clo}(S_{C})=\frac{1}{|V_{C}|}\sum\limits_{v \in V_{C}} C_{Clo}(v,S_{C}). $$
4. **Average eigenvector centrality:** Let *A*=(*a*_*i*,*j*_) be the adjacency matrix of *G*=(*V*,*E*), i.e., *A* is a symmetric |*V*|×|*V*| matrix with entry *a*_*i*,*j*_=1 if *v*_*i*,*j*_∈*E* and *a*_*i*,*j*_=0 otherwise. The *eigenvector centralityC*_*E*_ of a node *v*∈*V* is
  $$C_{E}(v,G) = \frac{1}{\lambda}\sum\limits_{w\in V}a_{w,v} C_{E}(w,G), $$
  where *λ* is the (absolute) largest eigenvalue of *A*. The *average eigenvector centralityC*_*E*_(*S*_*C*_) for a subgraph *S*_*C*_=(*V*_*C*_,*E*_*C*_) is defined as
  $$C_{E}(S_{C}) = \sum\limits_{v\in V_{C}} \frac{1} {|V_{C}|} C_{E}(v,S_{C}). $$
  Eigenvector centrality is based on the idea that importance of a node is determined by the importance of its neighbors: a node becomes more important the more important its neighbors are.
5. **Average clustering coefficient:** Let *N*_*v*_={*w*∈*V*:(*v*,*w*)∈*E*} be the set of all neighbors of a node *v*∈*V*. The local clustering coefficient of *v* is then defined as
  $$ C_{Clu}(v,G) = \frac{| \{e_{j,k} \in E : j, k \in N_{v} \} | }{ |N_{v}|(|N_{v}|-1)/2}. $$
  For a given subgraph *S*_*C*_=(*V*_*C*_,*E*_*C*_), we define the *average clustering coefficientC*_*Clu*_(*S*_*C*_) as the mean of *C*_*Clu*_(*v*,*S*_*C*_) over all *v*∈*V*_*C*_.
6. **Mean path length:** The *mean path length* for a subgraph *S*_*C*_=(*V*_*C*_,*E*_*C*_) is the average length of all shortest paths between all pairs of nodes *s*,*t*∈*V*_*C*_ in the graph *S*_*C*_:
  $$ C_{P}(S_{C}) =\frac{1}{|V_{C}|(|V_{C}|-1)} \sum_{s,t \in V_{C}} d(s,t|S_{C}), $$
  where *d*(*s*,*t*|*S*_*C*_) is the length of the shortest path between nodes *s* and *t* in the subgraph *S*_*C*_.

In addition to the network centrality measures above, we also used the following similarity coefficients to filter clusters:

1. **Dice similarity coefficient:** For any given node *v*∈*V* in a graph *G*, let ${E^{G}_{v}} := \{e_{v,w}\in E\}$ be the set of edges adjacent to *v*. The dice similarity coefficient of the edge sets ${E^{G}_{v}}$ and ${E^{G}_{w}}$ of two nodes *v*,*w*∈*V* is defined as
  $$ C_{DS}(v,w,G) = \frac{2 | {E^{G}_{v}} \bigcap {E^{G}_{w}}|}{|{E^{G}_{v}}|+|{E^{G}_{w}}|}. $$
  The average dice similarity of a subnetwork *S*_*C*_=(*V*_*C*_,*E*_*C*_), *V*_*C*_⊆*V*, is
  $$ C_{DS}(S_{C}) = \frac{2}{|V_{C}|(|V_{C}|-1)} \sum\limits_{v,w \in V_{C}}C_{DS}(v,w,S_{C}). $$
2. **Wang similarity coefficient:** This coefficient is biologically motivated and is based on similarity between gene ontology terms. Wang similarity takes the hierarchical structure of the GO graph into account by aggregating the information of ancestor terms when comparing two GO annotations [56]. Writing *C*_*G*_(*v*,*w*) for the Wang similarity between the GO annotations of nodes *v* and *w*, we compute the within-cluster similarity *C*_*G*_(*S*_*C*_) as the average Wang similarity *C*_*G*_(*v*,*w*) between all pairs of genes *v*,*w* in the subnetwork *S*_*C*_.

We note that a number of different measures have been proposed to compute the semantic similarity between two GO terms, for a comprehensive review see Pesquita et al. [57]. The choice of GO semantic similarity measure and a comparative evaluation of different measures are still subject to debate in the literature, as no gold standard exists, and different studies come to different conclusions [57]. The choice of similarity measure is therefore somewhat arbitrary and a matter of personal preferences. We opted for Wang similarity because of own good experiences with this coefficient in previous work, and because it is implemented in the GOSemSim package in R [58], which helped seamless integration into our analysis script. We note however that Wang similarity can easily be replaced by other semantic similarity measures in our analysis pipeline.

Filtering of clusters in **C**^*hit*^ was performed using the above topological and similarity measures as follows: We computed all topological and similarity measures for each subnetwork in **C**^*all*^, and performed a Wilcoxon test to assess differences of means of significantly enriched subnetworks in **C**^*hit*^ with randomly selected clusters in **C**^*all*^∖**C**^*hit*^ of the same size. Clusters that yielded a significant difference of the mean for all or all but one topological and semantic similarity measure at a significance level of 5% were considered for further analysis. By this, we ensure a stringent selection of subnetworks for further analysis: Resulting subnetworks are both enrichted with hits from the RNAi screens, and show topological properties that distinguish them from random clusters. In combination, these criteria resulted in a stringent selection of subnetworks, compare Table 1. We note that in theory, due to the variation of the cluster size parameter in ClusterONE, **C**^*hit*^ may contain clusters that are subsets/supersets of one another, however after filtering using the similarity and centrality measures we did not observe clusters that were subsets or supersets of other clusters in the analysis performed here.

**Table 1:** **P-values of Wilcoxon test to determine significance of mean values of network centralities and semantic measures for subnetwork**

	**HIV**		**HCV**		**Combined**		
**Centrality measure**	**s66**	**s52**	**s43**	**s64**	**s46**	**s52**	**s239**
Betweenness	< 0.0001	0.0131	0.0247	0.0005	0.0131	0.0131	0.0040
Closeness	<0.0001	0.0131	0.0247	0.0005	0.0131	0.0131	0.0040
Clustering Coefficient	< 0.0001	0.0247	0.0001	0.0005	0.0131	0.0131	0.0040
Eigenvector Centrality	< 0.0001	0.0131	1	1	1	0.0057	0.0057
Node Degree	< 0.0001	1	0.0247	0.0005	0.0211	0.0131	0.0040
Path Length	< 0.0001	0.0131	0.0247	0.0002	0.0131	0.0131	0.0040
Dice Similarity	< 0.0001	0.0131	0.0247	0.0005	0.0131	0.0131	0.0040
Wang Sim. (GO.BP)	0.0004	0.0286	0.5926	0.6009	0.0284	0.0286	0.0136
Wang Sim. (GO.CC)	0.0004	0.0286	0.5926	0.0315	0.0284	0.0286	0.0136
Wang Sim. (GO.MF)	0.0004	0.0286	0.0498	0.7713	1	0.3429	0.1077

A Wilcoxon test was used to determine the significance of network centrality measures and semantic similarity measures of subnetworks significantly enriched with RNAi screening hits. Average similarity measures over all nodes in a given enriched cluster were tested against non-enriched subnetworks of comparable size, using a Wilcoxon test to assess significance of the differences between the means for each of the given network centrality and semantic similarity measures. Shown are resulting p-values for two clusters for HIV, two clusters for HCV, and three combined clusters.

### Software and availability:

We implemented our data analysis pipeline in R [59]. Graph based calculations and reconstruction of subnetworks were performed using the iGraph library [60]. Network visualization was performed using Cytoscape [61]. All Reactome pathway and GO based enrichments were computed using the Bioconductor packages *clusterProfiler* and *ReactomePA* [62,63]. Semantic similarities were computed using the *GOSemSim* package [58]. R-code and data used are available on request from the authors.

## Results

Given the long and often largely non-overlapping hit lists from RNAi screens targeting viral infection, a central aim of our analysis was to select a small number of most significant, infection-relevant host protein subnetworks for further manual analysis, and thus to pick most promising candidates from the original screens for functional characterization. We are therefore interested in a small set of significant clusters, that are both enriched with hits from the RNAi screens, and play a central role in the host or virus-host protein interaction network.

We used RNAi data from seven different, published genome-wide RNAi screens focusing on the three viruses HIV [10,12,53], HCV [13,18,54] and WNV [11]. Hit lists from screens targeting the same virus were combined and analyzed in a virus-specific way, as well as all data pooled for pan-viral analysis of host restriction and host dependency factors. Data were analyze as described in Materials and methods and as illustrated in Figure 1. Analysis of the single West Nile virus screen did not yield significant results after filtering, probably due to too small number of hits included in the analysis. We did include this virus in the pan-viral analysis. Table 2 gives an overview over resulting hits for HIV-1 and HCV, discussed in more detail below.

**Table 2:** **Key results achieved for HIV-1 and HCV**

**Virus**	**Subnetwork**	**Predicted novel host factors**
HIV	HIV_s52	
		∙ KDM4B - lysine-specific demethylase 4B
	HIV_s66	
		∙ HNRNPK - Heterogeneous nuclear ribonucleoprotein K (hnRNP K) (Transformation up-regulated nuclear protein) (TUNP)
		∙ HNRNPL - Heterogeneous nuclear ribonucleoprotein L
		∙ HNRNPM - Heterogeneous nuclear ribonucleoprotein M
		∙ HNRNPU - Heterogeneous nuclear ribonucleoproteinU (hnRNP U) (Scaffold attachment factor A) (SAF-A) (p120) (pp120)
		∙ RBM11 - Splicing regulator RBM11 (RNA-binding motif protein 11)
		∙ RBM41 - RNA-binding protein 41 (RNA-binding motif protein 41)
		∙ RBM42 - RNA-binding protein 42 (RNA-binding motif protein 42)
		∙ RBM4B - RNA-binding protein 4B (RNA-binding motif protein 30) (RNA-binding motif protein 4B) (RNA-binding protein 30)
		∙ ‘RBM7 - RNA-binding protein 7 (RNA-binding motif protein 7)
		∙ SRSF3 - Serine/arginine-rich splicing factor 3 (PremRNA-splicing factor SRP20) (Splicing factor, arginine/serine-rich 3),
		∙ SRSF4 - Serine/arginine-rich splicing factor 4 (Pre-mRNA-splicing factor SRP75) (SRP001LB) (Splicing factor,
		arginine/serine-rich 4)
		∙ SRSF10 - Serine/arginine-rich splicing factor 10 (40 kDa SR-repressor protein)
HCV	HCV_s43	
		∙ *α* *β* Crystallin Complex subunits (CRYBAA, CRYBAB, CRYBA1, CRYBA2, CRYBA4, CRYBA1, CRYBB1, CRYBB2, CRYBB3)
		∙ Heat-shock proteins (HspB1, HspB2, HspB6, HspB7 and HspB8)
	HCV_s64	
		∙ Tyrosine-protein phosphatase non-receptors, various types (PTP-1B, TCPTP, PTP-H1, PTPase MEG2)
		∙ Tankyrase-1 (Poly-ADP-ribosyltransferase)

The table shows the main novel findings for HIV-1 and hepatitis C virus obtained by mapping RNAi data to protein interaction networks, and using the clustering and filtering procedure proposed here. Results for the combined analysis are given in Additional file 5.

### Human immunodeficiency virus-1 (HIV-1)

**Figure 2:**
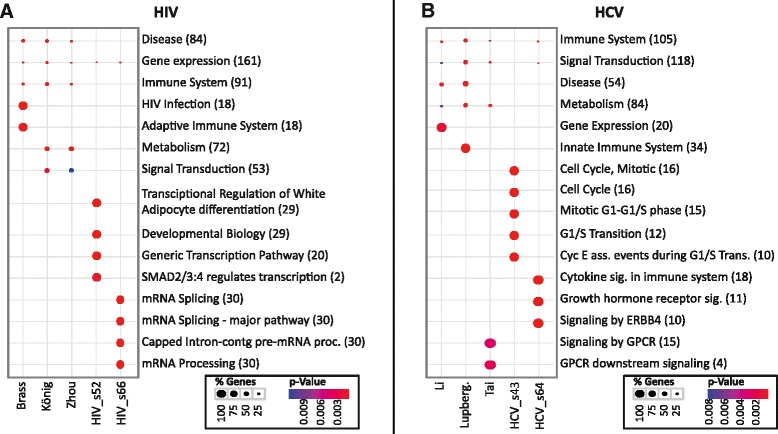
**HIV and HCV enrichment analysis.** The figure shows Reactome pathways annotations significantly enriched with hits from the individual RNAi screens or significant clusters from **(A)** HIV and **(B)** HCV. Size of the dots indicates percentage of genes in the respective annotation category that were significant in the screen, color codes statistical significance of enrichment.

Two significant subnetworks of size 52 (HIV_s52) and 66 proteins (HIV_s66), respectively, were obtained from analysis of the three HIV screens after filtering as described in Materials and methods. These subnetworks are shown in Additional file 1: Figure S1 and Additional file 2: Figure S2, respectively. A Reactome pathway enrichment analysis of the subnetworks as well as the original screens is shown in Figure 2A. The pathway analysis of the three screens individually yields the expected, albeit very general pathways, such as *Immune System*, *HIV Infection*, *Metabolism* or *Signal Transduction*. This is a typical outcome for geneset or pathway enrichment analysis with large hit lists from RNAi screens, which often results in very unspecific and general terms as the only significant outcomes. In contrast, due to the inclusion of protein neighborhoods and focusing on enriched subnetworks of the host protein network, much more specific results can be obtained using our approach, as illustrated for the HIV_s52 and HIV_s66 subnetworks (Figure 2A).

The HIV_s52 subnetwork consists primarily of genes involved in transcription, and comprises in particular subunits of the mediator complex. This complex is a transcriptional coactivator, involved in the regulation of expression of RNA polymerase II transcripts, and thus of all protein coding and most non-coding RNA genes [64]. The mediator complex has previously been identified in the context of HIV-1 infection in the meta-analysis by Bushman et al. [24] and was a major hit in the RNAi screens by Zhou et al. [53] and König et al. [12]. This discovery has led to different hypotheses about the role of the mediator complex in HIV infection. While Zhou et al. suggest that mediator complex subunits are required for Tat-activated transcription, König et al. speculate that the complex may be involved in reverse transcription. The exact role of the mediator complex in the HIV lifecycle still needs to be determined. Interestingly, transcriptional regulation does not show up in individual enrichment analysis of the screens by König et al. and Zhou et al. In contrast, it is highly significant for the HIV_s52 subnetwork, underlining the gain in power brought by a meta-analysis and by inclusion of protein neighborhoods in analyzing RNAi data (Figure 2).

The HIV_s66 subnetwork comprises many members of the heterogeneous nuclear ribonucleoprotein subunits (hnRNP) and serine/arginine rich splicing factors. The different hnRNP subunits participate in different steps in the RNA metabolism, including splicing, export, localization and translation [65]. Similarly, several of the serine/arginine rich splicing factors in the HIV_s66 subnetwork are known to have direct interactions with HIV viral proteins [66]. Correspondingly, enriched pathways in the HIV_s66 subnetwork are related to mRNA processing and splicing (Figure 2A). A recent study by Lund et al. focused on the hnRNP complexes, and mechanistic details of its involvement in HIV-1 infection [67]. The authors report that loss of the hnRNP A1 subunit increases the expression of HIV Gag and Env, but with no subsequent increase of viral RNA. In contrast, depletion of hnRNP A2 increases both Gag protein and HIV-1 RNA levels. Changes in expression of different isoforms of hnRNP D had very diverse effects, where some isoforms increased HIV-1 gene expression, whereas others brought the cells into a non-permissive state.

### Hepatitis C virus

We next repeated the analysis for the three hepatitis C virus screens by Li et al., Tai et al. and Lupberger et al. [13,18,54]. Combined analysis and submodule filtering as above resulted in two different subnetworks with 43 proteins (HCV_s43) and 64 proteins (HCV_s64), respectively, compare Additional file 3: Figure S3 and Additional file 4: Figure S4. Reactome enrichment showed that both modules were functionally very specific (Figure 2B).

The HCV_s43 module mainly contains dual specificity protein phosphatases, heat shock proteins (HSPs), crystalline proteins and mitogen-activated protein kinases (MAPKs). In particular the MAPKs are interesting, as they play a key role in cell growth and proliferation and are associated with hepatocellular carcinoma - the end stage of chronic HCV infection [68]. On the other hand, the HSPs and crystalline proteins both act as chaperones. Hsp72, one of the heat shock proteins in the HCV_s43 network, is known to be a positive regulator of HCV RNA replication by increasing replication complex levels [69]; furthermore, Lim et al. recently showed that the viral protein NS5A increases Hsp72 levels through the transcription factors HSF1 and NFAT5 [70], thus increasing its own replication. Reactome enrichment analysis of the HCV_s64 subnetwork shows enrichment in cytokine signaling, growth hormone receptor signaling, and ERBB4 signaling. The subnetwork in particular comprises several interleukin receptors and subunits, as well as insulin receptor and receptor substrate. The interleukins play an important role in suppression of infection, it is thus no surprise that HCV itself interacts with different interleukins to inhibit the cellular antiviral response [71-73].

### Pan-viral host factors

To get an overview over pan-viral host factors, we next pooled all seven screens (3 HIV, 3 HCV, 1 WNV) and analyzed the combined hit list [10-13,18,53,54]. Using our pipeline, we identified three highly significant subnetworks of size 46 proteins (Combi_s46), 52 proteins (Combi_s52) and a large network with 239 proteins (Combi_s239). The Combi_s52 network was identical to the one described for HIV, and is thus not discussed further here (see results on HIV).

**Figure 3:**
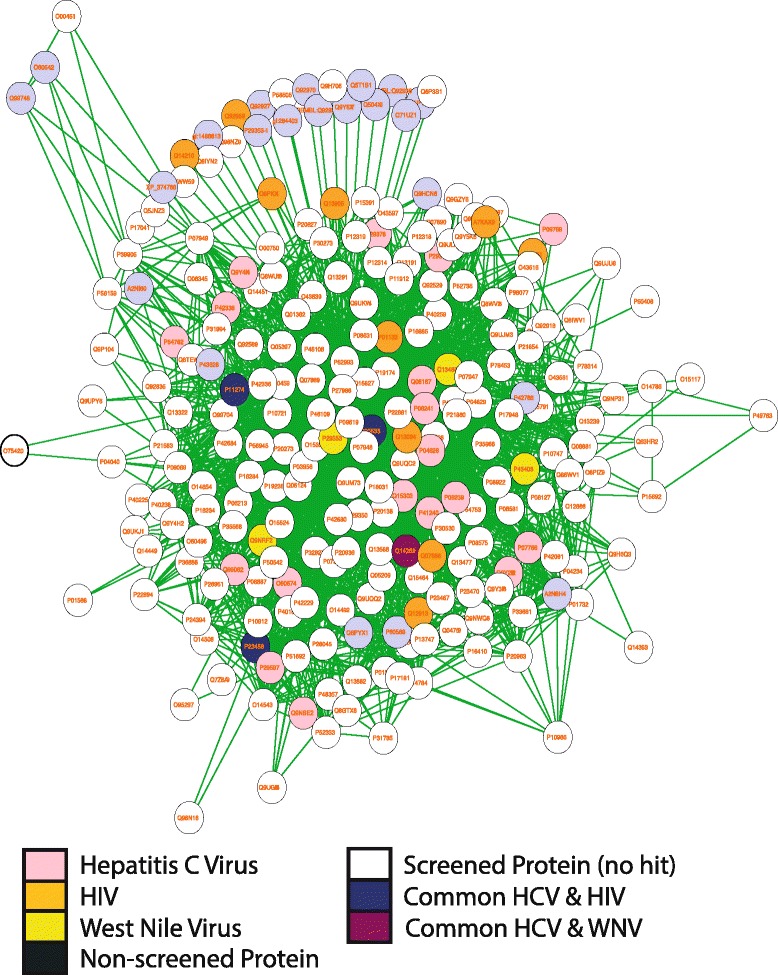
**Combi_s239 subnetwork- subnetwork resulting from analysis of all seven RNAi screens for three different viruses (HIV, HCV, WNV).** Nodes represents proteins and node labels represent Uniprot identifiers. All colored nodes represent hits from a RNAi screen, white nodes represent proteins from the Dharmacon library and black nodes are proteins from the Hu.PPI but not in the Dharmacon library.

The Combi_s239 subnetwork contains 17 tyrosine-protein kinases, 6 tyrosine-protein phosphatase non-receptors, 5 insulin receptor substrates, and an insulin receptor (see Additional file 5 and Figure 3). Indeed, insulin resistance is one of the effects observed in HCV infected patients as the disease progresses. A recent study identified components of the insulin signaling pathway that are altered by HCV, conferring insulin resistance in the patient [74]. The study showed that PTPB1, a tyrosine phosphatase, is significantly induced in infected cells. Supporting evidence also comes from a study by Garcia-Ruiz et al. who showed that insulin resistance is also associated with IFN- *α* resistance in Hep-G2 cells with increase PTPB activity [75]. Both these resistance types were lowered using Metformin, in both studies. The presence of several PTPBs in this network provides a basis for further experimentation with appropriate drugs that can keep the insulin-IFN- *α* resistances in check.

The Combi_s239 subnetwork furthermore contains several proteins from the Src kinase family. In WNV, it is known that e.g. c-Yes, a member of this family, is required for transportation of virions through the secretory pathway [76]. Several of the Src kinase family members are activated by HIV Nef [77], and also HCV NS5A induces phosphorylation events in the Src family [78-80].

The Combi_s46 subnetwork consists primarily of SMAD and zinc finger proteins. The SMADs are involved in TGF- *β* signaling, where they activate downstream gene expression [81,82]. TGF- *β* is an immunosuppressive cytokine, its modulation is therefore advantageous for parasitic viruses [83,84]. Indeed, HCV suppresses the TGF- *β* mediated transcriptional activation by the full-length polyprotein and NS3-viral proteins in a SMAD-R dependent manner [85]. Zinc finger proteins on the other hand have antiviral activity: Sakkhachornphop et al. have shown that a zinc-finger protein targets the 2-long terminal repeat (2-TLR) circle junctions of HIV-1 DNA [86,87]. This region of the HIV genome is cleaved by HIV integrase, and blocking this site restricts HIV-1 gene transcription.

### Mapping tissue-specific expression data

Given the filtered, significant subnetworks for the different viruses, we next addressed the problem to select suitable candidates for further experimental validation from the subnetworks, and thus ultimately possible targets for antiviral drugs. Of particular interest are proteins that are strongly expressed in tissues targeted by a given virus. Such tissue-specific or cell-line specific expression data is widely available through the Human Protein Atlas [88]. We overlaid subnetworks with tissue-specific expression data, and retained only proteins in the subnetwork that had moderate or high expression levels in the Protein Atlas database. Given the high rates of false negatives in RNAi screens [27], we do not necessarily require that candidate genes are direct hits in any of the screens.

**Figure 4:**
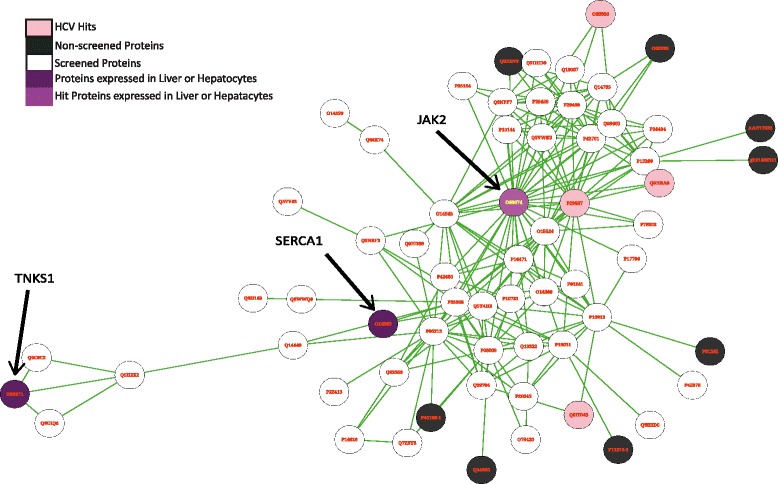
**The figure shows the HCV_s64 subnetwork, including TNKS1, SERCA1 and JAK2.** Tissue-specific expression data from the Human Protein Atlas were overlaid on the network using data from hepatocytes.

For hepatitis C virus, expression levels were selected from hepatocytes, resulting in three proteins that remained in the HCV-s64 subnetwork: Tankyrase-1 (TNKS1, also known as PARP5A, PARPL, TIN1 and TINF1), Sarcoplasmic/endoplasmic reticulum calcium ATPase 1 (SERCA1) and JAK2, compare Figure 4. Of these, TNKS1 and SERCA1 have not been reported as hits in any of the three HCV screens used. Interestingly, SERCA2, a close family member of SERCA1, has been shown to play an important role in HCV core induced ER stress and control of apoptosis [89]. As SERCA1 is closely interacting with SERCA2 and has similar functions, a similar role might be played by SERCA1 in HCV infection. TNKS1 on the other hand is involved in WNT signaling, regulation of telomere length, and vesicle trafficking. TNKS1 has previously been suggested as an attractive anti-cancer target [90], and is involved in HCV-induced apoptosis [91]. In case of HIV, we filtered proteins based on expression in macrophages. This resulted mainly in different subunits of the heterogeneous nuclear ribonucleoproteins (hnRNPs) as highly expressed putative antiviral targets.

## Discussion and conclusion

Genome wide RNAi screening experiments typically result in lists of hundreds of “hit” genes, and the selection of promising candidates for biochemical follow-up as well as their placement in the underlying molecular processes is a significant challenge [20]. To complicate matters further, in particular for viral RNAi screens, very low overlap has been reported even for screens targeting the same virus [24]. High false negative rates are likely a major contributing factor to this problem [27]. While geneset enrichment approaches can help to interpret lists of hit genes, they in our experience typically lead to very general, unspecific terms and often fail to achieve statistical significance for concrete, specific biological processes or pathways when applied to RNAi screening data. This problem clearly is aggravated if hit lists are prone to high levels of false negative results, and it is then a very challenging problem to pick interesting candidates for further experimental characterization.

In this work, we have developed a network-based approach for gene prioritization. The simple underlying idea is to interpret hit genes from RNAi screening experiments in their biological context, by taking the host cell protein-protein interaction (PPI) network into account. We cluster this PPI network to identify highly connected subnetworks, and then map the RNAi data onto this clustered network to find enriched submodules. Additional experimental data such as known virus-host interactions, gene expression data or e.g. proteomics data can easily be integrated at this stage and can be included in the network-based analysis. Similarly, it is straightforward to combine data from different screens for the same or even for different viruses at this level, to enable a network-based meta analysis of virus-host interactions. We exemplify this in a meta-analysis over seven different viral RNAi screens targeting three different viruses. In contrast to traditional geneset enrichment analysis, no prior definition of relevant gene sets (e.g. gene ontology annotations or biological pathways) is required, but instead gene sets are automatically defined by clustering of the PPI network. This is indeed an advantage and disadvantage at the same time: While we do not require a-priori defined gene sets for our analysis, our approach clearly depends on the underlying PPI network that must be given as input. Unfortunately, in particular for yeast-2-hybrid experiments, such networks are known to contain many false positive connections, which may negatively impact our analysis. Furthermore, we specifically opted to include high-confidence predicted interactions from the STRING database, which was required to obtain a sufficiently dense, connected network to permit further analysis. There is thus an inherent tradeoff between reliability of the underlying network used and sufficient network size and connectivity to allow a meaningful analysis. Similarly, the choice of clustering algorithm and similarity measures used to further filter significant networks will impact results. As proteins often perform multiple functions in a cell, we decided to use a clustering algorithm that allows for overlaps between different clusters, permitting individual proteins to be part of several different subnetworks. We furthermore performed our analysis with a whole range of parameters for the desired cluster size, using a redundant set of clusters of different sizes in the ensuing network centrality and similarity based filtering step. We thereby let the algorithm automatically select significant clusters of all sizes.

As no gold standard is available for virus-host interaction networks and RNAi screening data analysis, it is very difficult to assess the influence these different clustering parameters and false-positive or false-negative interactions in the underlying PPI network have on results. Reassuringly, our results show that we recover many of the known hits for the different viruses used in this study, and top candidates resulting from our gene prioritization approach are largely confirmed by other meta analysis approaches that have been performed using different methods. For example, Bushman et al. performed a meta-analysis of all published HIV-1 RNAi screens in 2009 [24], and also identified the mediator complex and hnRNPs as major HIV-1 host cell factors in their analysis. The mediator complex is also reported by Murali et al. in their analysis [38], whereas two further studies by Bader and Nepusz, respectively, identified the hnRNPs using MCODE, a different clustering algorithm than employed in our work [55,92]. Other related approaches include the work by MacPherson et al. [39], Dickerson et al. [30], Snijder et al. [37] and the VirHostNet database developed by Navratil et al. [93]. A unique aspect of our analysis is the comparative analysis over different viruses, with a specific focus on functional subnetworks in this pan-viral meta-analysis.

There are two further assumptions that we make in our analysis, that are worthy a brief discussion. The first, noncritical assumption we made in this manuscript concerns the expression analysis, overlaying the tissue specific expression data for hit selection onto the PPI network. We here made the assumption that low tissue expression of a gene implies that the gene is not a good target and was used as reason to exclude the gene from further consideration. We use this assumption here to filter genes within a subnetwork, but this is clearly a very crude approximation and many cases are conceivable where also a lowly expressed gene may be a very good drug target and may play an important role in infection. Obviously the inverse is not true: High expression alone does not make a gene a good target. The second assumption is critical: Our subnetwork analysis is based on the assumption that due to technical and biological variability, different genes within a subnetwork may be identified in different screens, but that indeed the entire subnetwork or sub-complex is a relevant host factor. In particular in light of high false negative rates in RNAi screens [27] and further variability due to e.g. different experimental protocols, cell lines and viral genotypes used and different transfection and infection times, it is very plausible that different genes in the same pathway or subnetwork will be identified in different screens, even when targeting the same virus. Our further subnetwork analysis therefore requires that subnetworks resulting from the clustering have high functional consistency, in the sense that the proteins within one cluster need to be involved in the same biological process or pathway, whereas different clusters should be functionally distinct – this is a *conditio sine qua non* when speaking of significance of a subnetwork. In line with this, the identification of putative targets in our analysis focuses on all proteins in a subnetwork, even if they did not show up as hits in any of the original screens considered. Before proceeding with such hits in a drug development pipeline, clearly additional experiments are required to confirm a role of these hits in the infection process, and in particular an effect of targeting the candidate gene on viral infection. As cells have many redundant mechanisms, even if a host gene is involved in viral infection, targeting this gene may not be sufficient to inhibit viral replication. Detailed mathematical modeling of the underlying processes in the subnetwork may then be a good option to identify optimal treatment strategies, but goes beyond the scope of the present work [94].

While we have developed the approach presented in this manuscript for the analysis of viral RNAi screening data, the general pipeline is applicable to any type of experiment resulting in long “hit” gene lists. Examples include gene expression data e.g. from microarray or transcriptome sequencing experiments, methylation profiles, genomic data such as array CGH or DNA sequencing, and proteomic assays based on mass spectrometry or protein arrays. Similarly, biological questions addressable with our pipeline extend well beyond viral infection, and basically include any assay where a mechanistic biological understanding is sought for based on large-scale, high-throughput data sets. In particular with the current developments in and increasing availability of big data in biology, network-based analysis approaches are a fundamental tool to interpret and understand the underlying biological processes, and will become more and more important as available data grows. We demonstrate the use of such network-based analysis methods on the concrete example of virus-host interactions in the present work.

## Additional files

Additional file 1
**Figure S1.** HIV_s52 subnetwork: The figure shows the HIV_s52 subnetwork resulting from the analysis of the HIV screens. The subnetwork primarily consists of genes involved in transcription, and particularly comprises the mediator complex.

Additional file 2
**Figure S2.** HIV_s66 subnetwork: Shown is the HIV_s66 subnetwork resulting from the HIV screen analysis. The network essentially contains splicing factors and members of the hnRNP complex.

Additional file 3
**Figure S3.** HCV_s43 subnetwork: This subnetwork from the analysis of the three HCV screens comprises mainly heat shock proteins and proteins of the MAPK pathway.

Additional file 4
**Figure S4.** HCV_s64 subnetwork: The HCV_s64 subnetwork is one of two significant subnetworks for the HCV screens, and contains interleukin receptors, cytokines and growth hormone receptors.

Additional file 5
**List of proteins in Combined_s239 subnetwork.** This xls file contains the proteins involved in the Combined_s239 network, together with additional annotation.
