# Supplementary figures and images for: An integrative approach for a network based meta-analysis of viral RNAi screens
Source: Algorithms Mol Biol. 2015 Feb 13;10:6. doi: 10.1186/s13015-015-0035-7 (PMC4331137; doi:10.1186/s13015-015-0035-7)

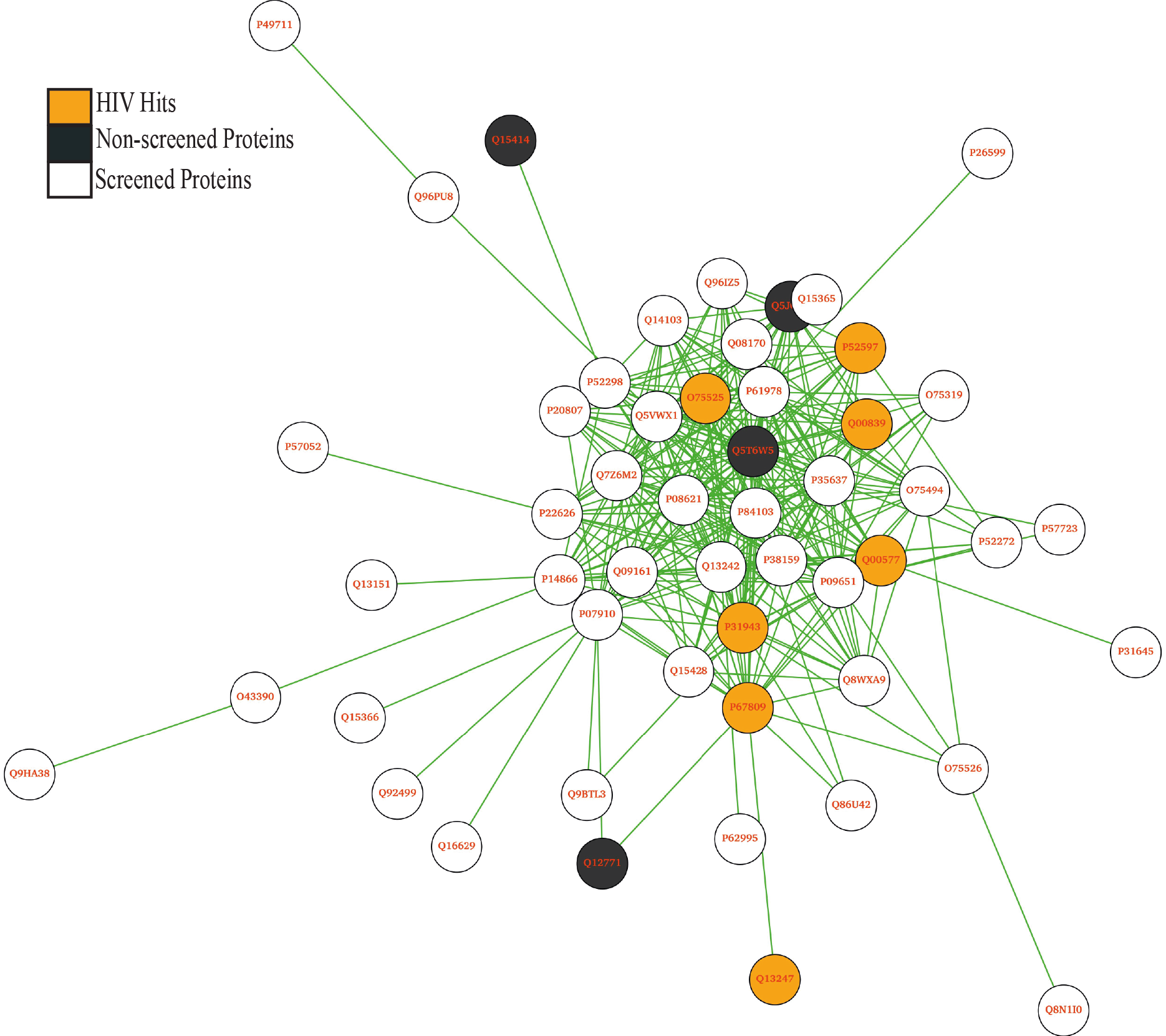

Supplement: Additional file 1 — Figure S1. HIV_s52 subnetwork: The figure shows the HIV_s52 subnetwork resulting from the analysis of the HIV screens. The subnetwork primarily consists of genes involved in transcription, and particularly comprises the mediator complex. [file 13015_2015_35_MOESM1_ESM.png]

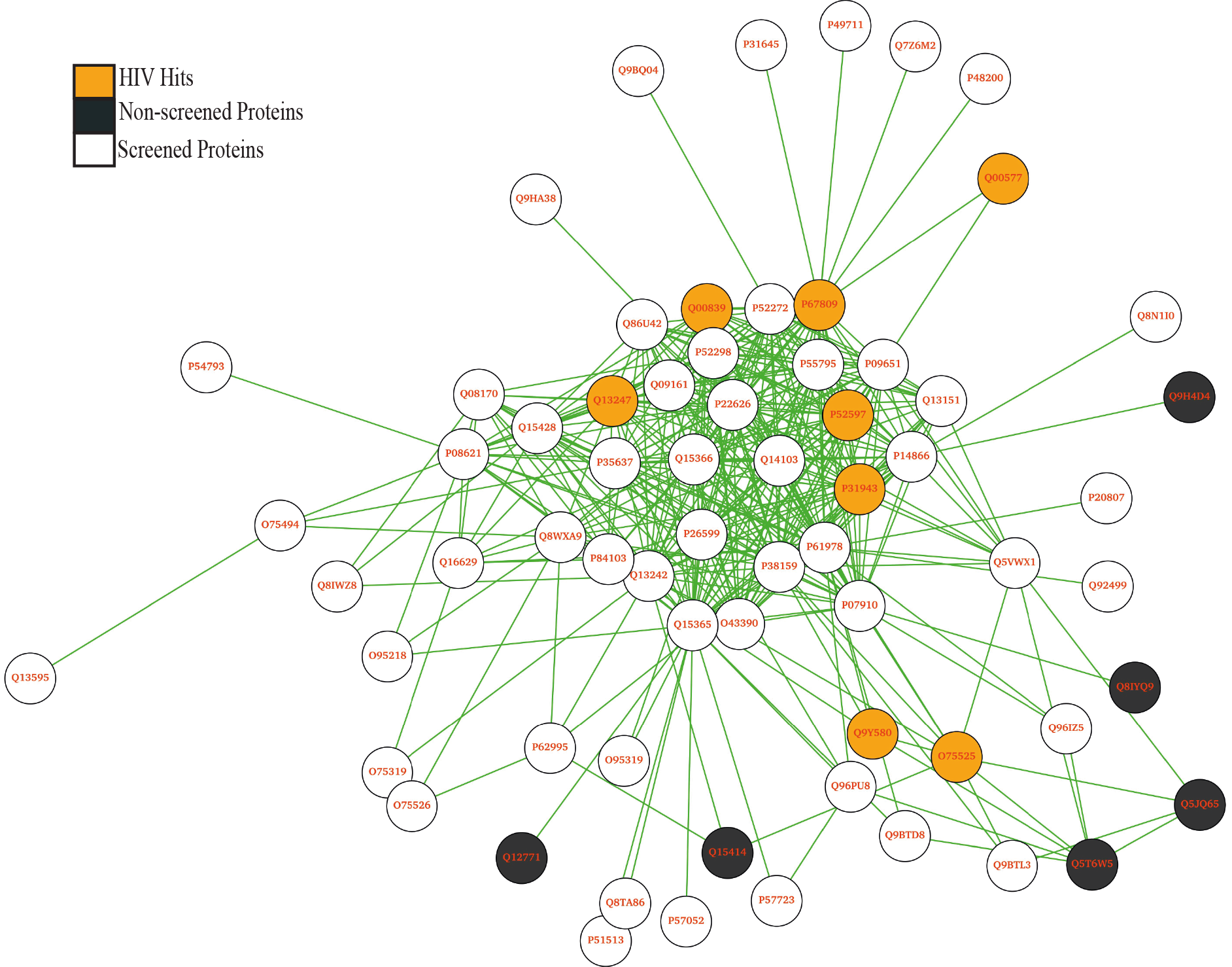

Supplement: Additional file 2 — Figure S2. HIV_s66 subnetwork: Shown is the HIV_s66 subnetwork resulting from the HIV screen analysis. The network essentially contains splicing factors and members of the hnRNP complex. [file 13015_2015_35_MOESM2_ESM.png]

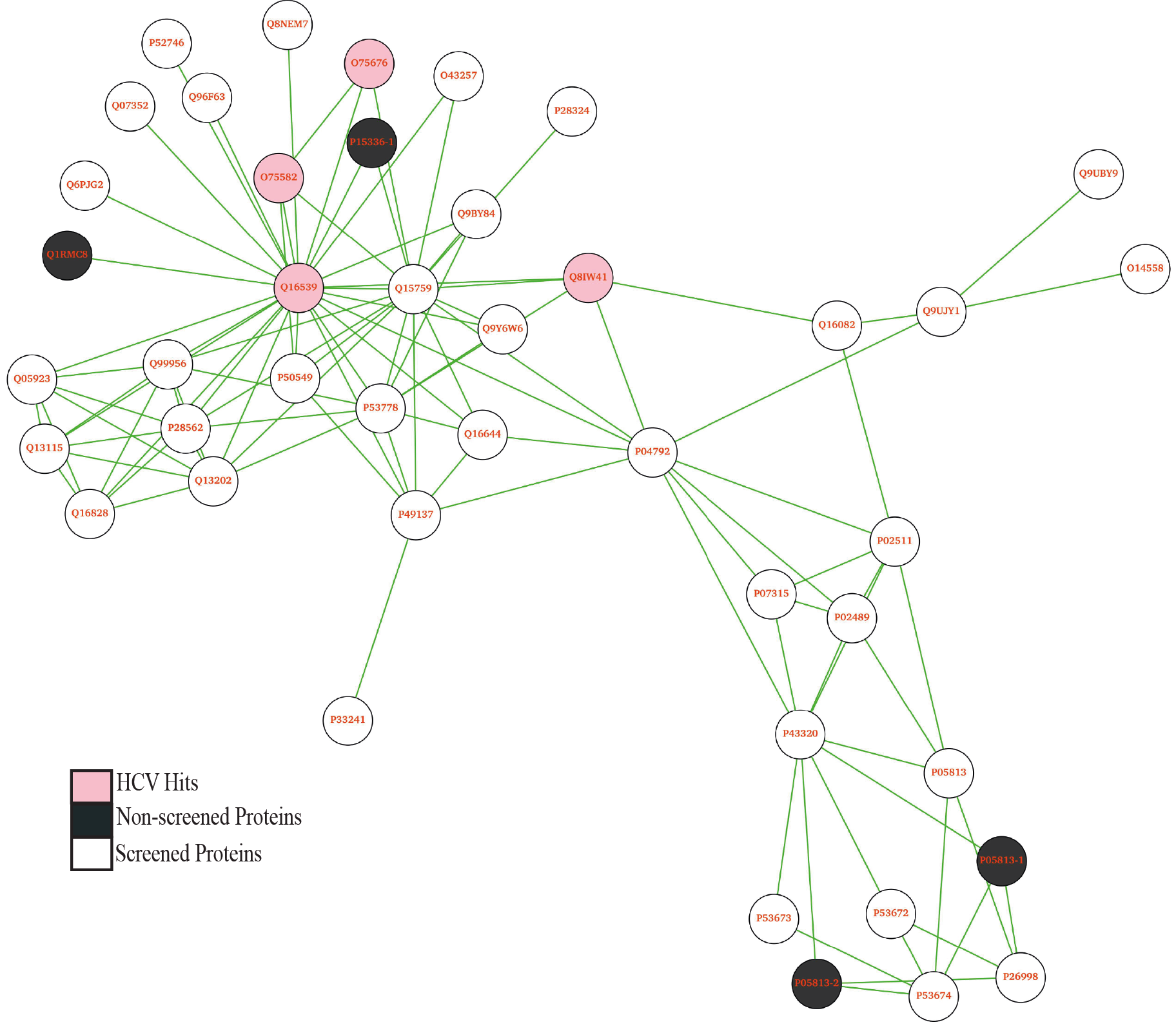

Supplement: Additional file 3 — Figure S3. HCV_s43 subnetwork: This subnetwork from the analysis of the three HCV screens comprises mainly heat shock proteins and proteins of the MAPK pathway. [file 13015_2015_35_MOESM3_ESM.png]

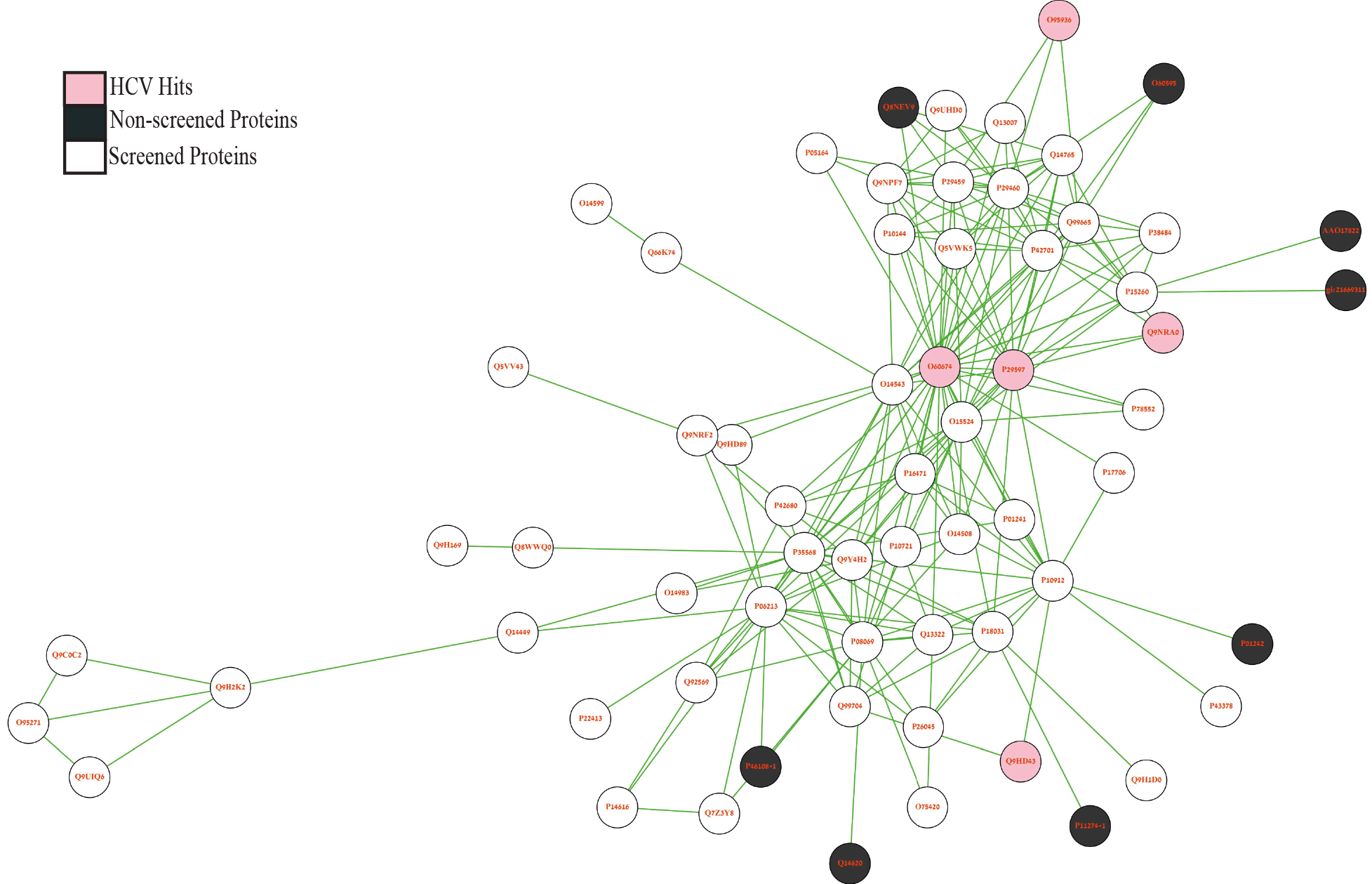

Supplement: Additional file 4 — Figure S4. HCV_s64 subnetwork: The HCV_s64 subnetwork is one of two significant subnetworks for the HCV screens, and contains interleukin receptors, cytokines and growth hormone receptors. [file 13015_2015_35_MOESM4_ESM.png]
